# Impact of Psoas Muscle Area Index on Short- and Mid-Term Mortality in Patients Undergoing Valve Surgery for Infective Endocarditis: A Retrospective Analysis

**DOI:** 10.3390/diagnostics14202259

**Published:** 2024-10-10

**Authors:** Christian Dinges, Matthias Hammerer, Nikolaos Schörghofer, Christoph Knapitsch, Gretha Hecke, Sophie Klaus, Johannes Steindl, Richard Rezar, Rainald Seitelberger, Uta C. Hoppe, Klaus Hergan, Elke Boxhammer, Bernhard Scharinger

**Affiliations:** 1 Department of Cardiovascular and Endovascular Surgery, Paracelsus Medical University of Salzburg, 5020 Salzburg, Austria; c.dinges@salk.at (C.D.);; 2 Department of Internal Medicine II, Division of Cardiology, Paracelsus Medical University of Salzburg, 5020 Salzburg, Austria; 3 Department of Radiology, Paracelsus Medical University of Salzburg, 5020 Salzburg, Austriabe.scharinger@salk.at (B.S.)

**Keywords:** computed tomography, endocarditis, psoas muscle area index, sarcopenia, valve surgery

## Abstract

**Background:** Sarcopenia, characterized by the loss of skeletal muscle mass, is an emerging comorbidity associated with poor outcomes in cardiovascular surgery. Its impact on mortality in patients undergoing valve surgery for infective endocarditis (IE) remains underexplored. This study investigates the relationship between sarcopenia, measured by the Psoas muscle area index (PMAi), and mortality in patients with IE undergoing valve surgery. **Materials and Methods:** We retrospectively analyzed 68 patients with IE who underwent valve surgery at a tertiary care center from 2013 to 2021. Sarcopenia was defined as being in the lowest quartile of PMAi, measured via preoperative computed tomography (CT). Baseline characteristics, survival outcomes, and factors influencing mortality were analyzed using Kaplan–Meier survival curves and Cox proportional hazards regression. The predictive value of PMAi for 1-year and 3-year mortality was assessed via receiver operating characteristic (ROC) curves. **Results:** Sarcopenia was strongly associated with increased mortality at both 1-year (HR: 0.378, *p* = 0.010) and 3-year follow-ups (HR: 0.457, *p* = 0.012). Female sex (OR: 275.748, *p* < 0.001) and older age (OR: 9.995, *p* = 0.003) were significant predictors of sarcopenia. Chronic kidney insufficiency (CKI) and the use of heart failure medication therapy also significantly impacted survival outcomes. **Conclusions:** Sarcopenia is a strong independent predictor of short- and mid-term mortality in patients undergoing valve surgery for IE. Routine radiological assessment of sarcopenia using PMAi could improve risk stratification and guide preoperative interventions. Tailored management strategies, especially in older women and patients with CKI, may enhance outcomes in this high-risk population.

## 1. Introduction

Infective endocarditis (IE) remains a serious condition associated with high morbidity and mortality despite advances in medical and surgical treatments [1]. This disease, characterized by infection of the endocardial surface of the heart, predominantly affects the heart valves and can lead to severe complications, including heart failure, systemic embolization, and persistent infection [2,3]. While the incidence of IE varies geographically, it remains a significant health burden, particularly among older adults and those with predisposing conditions such as prosthetic heart valves, congenital heart disease, and intravenous drug use [4,5]. Surgical intervention is often required in patients with IE, especially in cases complicated by heart failure, uncontrolled infection, or large vegetations. However, the decision to operate is complex and influenced by a multitude of factors, including the patient’s overall health status and comorbidities [6,7].

One emerging factor of interest in the management of IE is sarcopenia, a syndrome characterized by the progressive and generalized loss of skeletal muscle mass and strength. Sarcopenia is increasingly recognized as a critical determinant of outcomes in various medical and surgical conditions [8,9]. In the context of cardiovascular diseases [10], sarcopenia has been associated with poor outcomes in patients undergoing cardiac surgery [11], including those with heart failure [12] and coronary artery disease [13]. The role of sarcopenia in patients with IE, particularly those undergoing surgery, however, remains underexplored.

Sarcopenia is particularly relevant in patients with IE because the condition frequently affects older adults, a population already at high risk for muscle wasting. Moreover, the catabolic state induced by chronic infection, along with the potential for prolonged immobilization and nutritional deficiencies during the course of the disease, may further exacerbate muscle loss in these patients. This loss of muscle mass and function may impact not only the immediate perioperative outcomes but also long-term survival and recovery, as sarcopenia has been linked to increased frailty, reduced functional capacity, and higher rates of postoperative complications [14,15].

The psoas muscle, a major muscle of the lower back and a key indicator of overall muscle mass, has been increasingly utilized in research as a surrogate marker for sarcopenia [16]. Measurement of the psoas muscle cross-sectional area on computed tomography (CT) scans, normalized to the patient’s body surface area (BSA), has been shown to be a potential predictor of surgical outcomes across various patient populations. In patients undergoing major surgeries, including cardiac procedures, a reduced psoas muscle area (PMA) has been associated with increased morbidity and mortality [17,18]. Given the high stakes involved in the surgical management of IE, evaluating the Psoas area could provide valuable insights into patient risk stratification and decision-making processes.

Despite the potential significance of sarcopenia in influencing outcomes in IE patients, this factor is not yet routinely considered in clinical practice. Current guidelines for the management of IE [19] primarily focus on the timing and indications for surgery, the choice of antimicrobial therapy, and the management of complications, with limited emphasis on the patient’s nutritional and functional status. Incorporating sarcopenia assessment into the preoperative evaluation could enhance the ability to identify high-risk patients who may benefit from tailored interventions, such as prehabilitation or postoperative rehabilitation programs, to improve their muscle mass and strength before and after surgery.

This study aims to explore the relationship between sarcopenia, as measured by the psoas muscle area index (PMAi; psoas muscle area normalized to BSA), and outcomes in patients with IE undergoing surgery. Specifically, we will examine the association between the PMAi and short-term as well as mid-term survival in this patient population. By investigating this relationship, we hope to provide insights that could lead to more individualized and potentially more effective management strategies for patients with IE, particularly those who are elderly or have other risk factors for sarcopenia.

## 2. Materials and Methods

### 2.1. Study Population

This study included a consecutive series of 68 patients diagnosed with infective endocarditis who underwent valve surgery at a large tertiary care center in Salzburg, Austria, over a nine-year period from 2013 to 2021. Patients were identified using the hospital’s electronic medical records system, which includes all admissions and surgical procedures performed at the institution. Endocarditis diagnoses were confirmed according to the Duke criteria, which were applied by the treating clinicians at the time of hospital admission. A retrospective review of the medical records was conducted to verify the diagnosis and eligibility for inclusion in the study.

Adult patients diagnosed with endocarditis and determined to be suitable candidates for valve surgery by the multidisciplinary endocarditis team were eligible for inclusion. Patients were excluded if they had preoperative kidney failure requiring dialysis, were younger than 18 years of age, or had incomplete medical records. Additionally, patients were excluded if they lacked preoperative CT imaging (either no CT was performed, or the CT was conducted at an external facility without accessible data for analysis), or if the available CT images did not adequately cover the anatomical region up to the psoas muscle, making accurate PMA assessment impossible. All data were collected and analyzed retrospectively using electronic health records and radiological imaging archives.

### 2.2. Ethical Considerations

The study protocol was reviewed and approved by the Ethics Commission of the State of Salzburg (Ethics Approval Number: 1109/2023; date of approval: 29/9/2023). The research was conducted in compliance with the principles outlined in the Declaration of Helsinki and adhered to Good Clinical Practice guidelines. Given the retrospective nature of the study, the Ethics Commission granted a waiver of informed consent.

### 2.3. Endocarditis Diagnosis

All participants in this study were diagnosed with infective endocarditis according to the Duke criteria applicable at the time of their surgical evaluation. These criteria served as the standard for diagnosing endocarditis and determining the need for surgical intervention.

### 2.4. Indications for Valve Surgery

The decision to proceed with valve surgery for endocarditis was made by a multidisciplinary endocarditis team, which included cardiologists, cardiac surgeons, and infectious disease specialists. This team-based approach ensured that each patient’s surgical indications were thoroughly evaluated in accordance with established guidelines and best practices.

### 2.5. Surgical Procedure for Valve Replacement

For patients requiring valve replacement due to endocarditis, surgery was performed under general anesthesia. The procedure began with a median sternotomy to provide access to the heart. A cardiopulmonary bypass was initiated to maintain systemic circulation while the heart was temporarily stopped. Cardioplegia was administered to arrest the heart and protect the myocardium during surgery. The aorta was clamped to isolate the heart from the systemic circulation. The infected valve was carefully excised, with thorough debridement of all infected and necrotic tissue to reduce the risk of recurrent infection. A prosthetic valve or homograft, selected based on the patient’s specific anatomical and physiological requirements, was then implanted. The surgical field was meticulously inspected to ensure the complete removal of infectious material before the chest was closed. Postoperatively, patients were monitored closely for any complications and antimicrobial therapy was continued to ensure the full eradication of the infection.

### 2.6. CT Protocol and Measurement of PMA

All patients included in the study routinely underwent a non-contrast CT of the brain and a contrast-enhanced CT scan of the chest, abdomen, and pelvis using intravenous contrast during the arterial and portal venous phases. These scans were performed preoperatively to evaluate for embolic infarcts and both calcified and non-calcified plaques in the ascending aorta. The CT imaging was conducted with second-generation multidetector CT scanners, specifically 256- or 128-slice dual-source scanners (Revolution, General Electric Healthcare, IL, USA or Somatom Definition AS+, Siemens Healthcare, Erlangen, Germany). The tube voltage was adjusted based on patient size (ranging from 80–120 kVp) with active tube current modulation. A bolus-tracking technique was used, with 100 mL of non-ionic iodinated contrast media followed by 70 mL of saline solution, administered at a flow rate of 3.5–5 mL/s.

**Figure 1:**
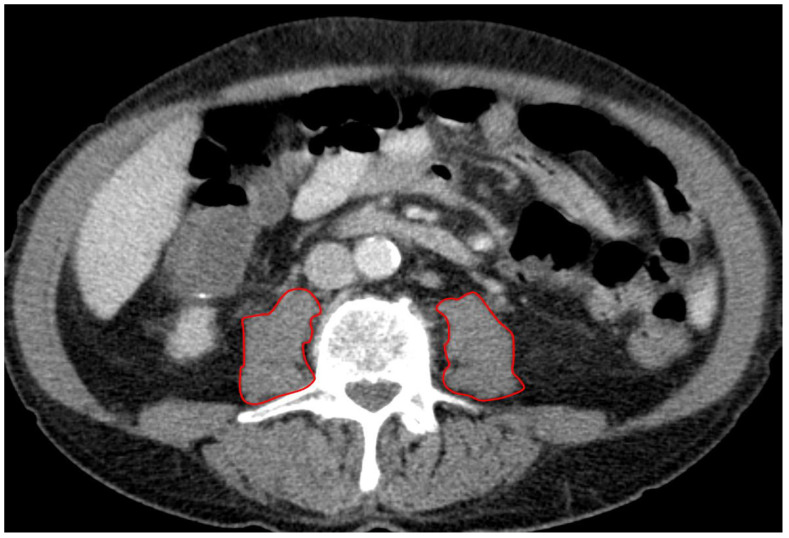
Radiological measurement of PMA. Figure 1 demonstrates the measurement of left and right PMA by manually tracing the perimeter of the psoas muscles on an axial CT scan. The combined areas of the left and right muscles, adjusted for BSA, provided the PMAi.

A board-certified radiologist blinded to all clinical data assessed the PMA (mm^2^) using a stationary workstation with dedicated software (Deep Unity Diagnostics 1.1.1.1, Dedalus Healthcare, Milan, Italy). Measurements were obtained from axial CT scans of the abdomen and pelvis during the portal venous phase, using a soft tissue kernel with a slice thickness of 3 mm and a reconstruction interval of 2 mm. The PMA was assessed in all patients at the level of the superior endplate of the third lumbar vertebra (L3) by manually tracing the right and left psoas muscles (Figure 1). The total PMA was calculated by adding the areas of both the left and right psoas muscles. Furthermore, the total PMA was adjusted for the patient’s body surface area (BSA), which was calculated using the Dubois formula: BSA = 0.007184 × height^^0.725^ × weight^^0.425^ (BSA: m^2^). This produced the indexed PMA (PMAi: mm^2^/m^2^).

### 2.7. Statistical Analysis

The statistical analysis and graphical representations were conducted using IBM SPSS Statistics, Version 25.0 (IBM Corp., Armonk, NY, USA).

A Pearson correlation analysis was conducted to examine the relationship between PMA and body constitution factors. In our study, PMA (measured in mm^2^) showed a stronger correlation with BSA, calculated using the Dubois formula (r = 0.548), compared to its correlation with body mass index (BMI, measured in kg/m^2^) (r = 0.189). Based on these findings, PMA was normalized by dividing it by BSA, resulting in an indexed PMA (PMAi: mm^2^/m^2^). Patients were then categorized into quartiles based on their PMAi, with those in the lowest quartile (Q1) classified as sarcopenic.

Expressions of baseline characteristics were statistically compared based on the two groups “sarcopenia” vs. “no sarcopenia“. The Kolmogorov–Smirnov–Lilliefors test was utilized to evaluate the normality of the data distribution. For normally distributed variables, results were expressed as mean ± standard deviation (SD) and compared using the unpaired Student’s *t*-test. In cases where the data were not normally distributed, the median and interquartile range (IQR) were reported, and the Mann–Whitney U test was used for statistical comparisons. Categorical variables were presented as frequencies and percentages, with comparisons made using the chi-square test.

A Kaplan–Meier survival curve, accompanied by a log-rank test, was created to assess differences in short- and mid-term survival between patients with radiologically confirmed sarcopenia and those without radiological evidence of sarcopenia. Additionally, survival outcomes were compared across the respective quartiles of the patient population.

For the assessment of 1-year and 3-year mortality, Cox proportional hazards regression models were employed to evaluate the relationship between sarcopenia, as indicated by PMAi, and survival outcomes. Prior to analysis, continuous variables were Z-transformed to standardize the data and allow for better comparability between variables. Univariate Cox regression analyses were performed for each potential predictor variable, with a significance threshold set at *p* ≤ 0.100 to identify candidate variables for inclusion in the multivariable model. Variables meeting this threshold were then included in a multivariable Cox regression model to adjust for potential confounders and assess the independent impact of sarcopenia on mortality risk. The proportionality assumption was verified, and hazard ratios (HR) with 95% confidence intervals (CI) were reported.

Additionally, receiver operating characteristic (ROC) curve analyses were performed to evaluate the predictive ability of PMAi and other clinical factors for 30-day, 90-day, 1-year, 2-year, and 3-year mortality. The area under the ROC curve (AUROC) was calculated to quantify the model’s ability to discriminate between survivors and non-survivors over these time frames. The optimal PMAi cut-off value for each time point was identified using the Youden index (YI), which selects the threshold that provides the best balance between sensitivity and specificity.

To identify potential factors influencing the presence of sarcopenia, an initial univariate binary logistic regression analysis was conducted. Metric variables were again standardized using Z-transformation to improve comparability. Following this, a multivariable binary logistic regression was performed to determine independent predictors of sarcopenia. Variables that showed an association with sarcopenia in the univariate analysis (*p* ≤ 0.100) were included in the multivariable model. A backward elimination method was then applied to remove non-significant variables, refining the model to retain only the most relevant predictors.

A *p*-value (two-tailed test) < 0.050 was considered statistically significant.

## 3. Results

### 3.1. Sarcopenia Based on PMAi Quartiles

To better define sarcopenia in the study population, PMAi was divided into quartiles. This stratification provides a clear framework for understanding the impact of sarcopenia on patient outcomes following valve surgery for endocarditis.

In Table 1, the PMAi quartiles are detailed as follows: patients in the first quartile (Q1) had a PMAi of less than 620.96 mm^2^/m^2^, indicating the most severe sarcopenia. Q2 ranged from 620.96 to 789.60 mm^2^/m^2^, Q3 from 789.60 to 940.91 mm^2^/m^2^, and Q4 included values greater than 940.91 mm^2^/m^2^. Patients in the lowest quartile (Q1) were identified as sarcopenic and demonstrated significantly worse survival outcomes compared to those in the higher quartiles (Q2–Q4).

### 3.2. Baseline Characteristics of Overall Study Cohort and Sarcopenic vs. Non-Sarcopenic Patients

Table 2 presents the baseline characteristics of 68 patients undergoing valve surgery for endocarditis, stratified by the presence or absence of sarcopenia. A baseline characteristics overview with a distinction between the sexes is provided as Supplementary Table S1. Sarcopenia was observed in 25% of the cohort (lowest quartile of PMAi = 17 patients), with the following significant differences between the sarcopenic and non-sarcopenic groups.

Female patients were more likely to be sarcopenic (82.4%, *p* < 0.001). Sarcopenic patients also had a higher mean age (68.1 ± 10.7 vs. 60.5 ± 11.7 years, *p* = 0.021) and lower body surface area (BSA), height, and weight (all *p* < 0.001). PMA without indexing was significantly lower in sarcopenic patients (*p* < 0.001), highlighting their reduced muscle mass. In terms of comorbidities, sarcopenic patients had a higher prevalence of coronary vessel disease (CVD) (*p* = 0.003).

There were no significant differences in operative factors, such as surgery, clamping, or perfusion time, nor in most pre-existing or postoperative conditions between the groups.

### 3.3. Kaplan–Meier Survival Curves Stratified by PMAi Quartiles and Sarcopenia Status

**Figure 2:**
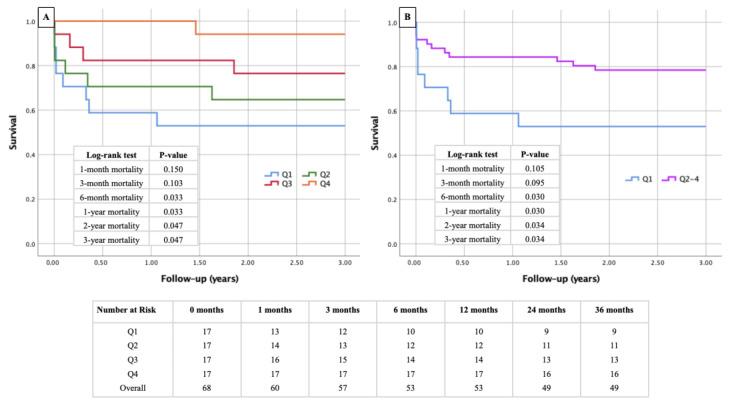
Kaplan–Meier curves with corresponding numbers at risk and log-rank tests for detection of short- and mid-term mortality regarding the presence or absence of sarcopenia. Figure 2 demonstrates Kaplan–Meier survival curves with a follow-up survival of 3 years post-surgery. In (**A**), all four quartiles of PMAi are demonstrated, whereas in (**B**), only the lowest quartile (defined as sarcopenic) was compared to the other non-sarcopenic quartiles.

The first Kaplan–Meier curve (Figure 2A) shows survival probabilities for patients divided into quartiles according to their PMAi. The lowest quartile (Q1), representing the most sarcopenic patients, consistently demonstrates lower survival rates compared to the higher quartiles (Q2, Q3, and Q4). The log-rank test indicates significant differences in survival between the quartiles, particularly at 6 months (*p* = 0.030), 1 year (*p* = 0.030), 2 years (*p* = 0.034), and 3 years (*p* = 0.034). The second Kaplan–Meier (Figure 2B) curve compares survival between patients with radiologically confirmed sarcopenia (PMAi in the lowest quartile) and those without sarcopenia (patients in the higher quartiles). The results again demonstrate poorer survival outcomes in the sarcopenic group, with significant differences emerging during follow-up, particularly in the mid-term periods. This underscores the impact of reduced PMAi on survival, with patients in the lowest quartile (= sarcopenia) showing a notably higher mortality risk.

### 3.4. Predictive Value of PMAi for Short- and Mid-Term Mortality in Endocarditis Patients

**Figure 3:**
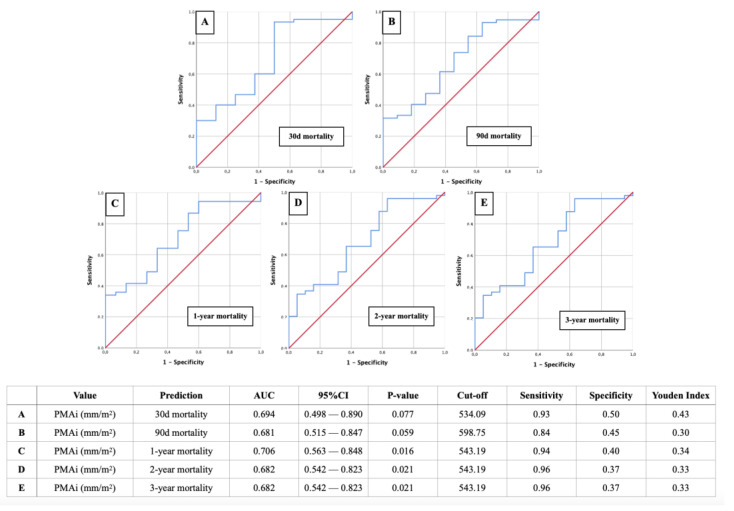
ROC curves for PMAi as a predictor of mortality at different time points. Figure 3 displays ROC curves assessing the predictive value of PMAi for mortality at 30 days (**A**), 90 days (**B**), 1 year (**C**), 2 years (**D**), and 3 years (**E**) post-surgery. The AUC values demonstrate the discriminatory ability of PMAi for predicting mortality at each time point. The corresponding cut-off values, sensitivity, specificity, and Youden index are presented in the accompanying table, highlighting the predictive performance of PMAi across short- and mid-term outcomes in endocarditis patients. Red line = reference curve; blue line = probability curve with AUC representing the degree/measure of separability.

The ROC curves of Figure 3 show the predictive ability of PMAi for mortality in patients with infective endocarditis following valve surgery. PMAi demonstrated moderate predictive power for 30-day, 90-day, 1-year, 2-year, and 3-year mortality, with AUC values ranging from 0.681 to 0.706. The predictive accuracy improved for mid-term mortality, with statistically significant results for 1-, 2-, and 3-year mortality (*p* < 0.05). Sensitivity was high across all time points, particularly for mid-term mortality (94–96%), though specificity remained moderate. A PMAi cut-off of approximately 543 mm^2^/m^2^ was identified as a key threshold for predicting mortality risk.

### 3.5. Cox Regression Analysis in Patients Undergoing Valve Surgery for Endocarditis

The Cox regression analysis in Table 3 explores the predictors of 1-year and 3-year mortality in patients undergoing valve surgery for infective endocarditis.

For 1-year mortality, the univariate analysis identified sarcopenia (as measured by PMAi) as a significant predictor of mortality (HR: 0.494, 95% CI: 0.265–0.920, *p* = 0.026). Similarly, chronic kidney injury (CKI) showed a strong association with increased mortality risk (HR: 5.503, 95% CI: 1.867–16.222, *p* = 0.002). In the multivariable analysis, both sarcopenia and CKI remained significant predictors, with PMAi continuing to indicate a reduced risk of mortality (HR: 0.378, 95% CI: 0.179–0.795, *p* = 0.010) and CKI showing elevated mortality risk (HR: 4.762, 95% CI: 1.452–15.621, *p* = 0.010). Furthermore, angiotensin-converting enzyme (ACE) inhibitors, angiotensin receptor blockers (ARB), and angiotensin receptor-neprilysin inhibitors (ARNI) (abbreviation: ACEI/ARB/ARNI) therapy emerged as a significant protective factor in the multivariable model (HR: 0.112, 95% CI: 0.014–0.870, *p* = 0.036), indicating a lower risk of death for patients receiving these medications.

For 3-year mortality, sarcopenia remained a significant factor in the univariate analysis (HR: 0.541, 95% CI: 0.317–0.925, *p* = 0.025), along with CKI (HR: 4.224, 95% CI: 1.510–11.818, *p* = 0.006) and the use of diuretics (HR: 3.133, 95% CI: 1.189–8.253, *p* = 0.021). In the multivariable analysis, sarcopenia maintained its independent association with increased mortality risk (HR: 0.457, 95% CI: 0.249–0.841, *p* = 0.012), as did CKI (HR: 3.097, 95% CI: 1.019–9.409, *p* = 0.046) and diuretics (HR: 2.846, 95% CI: 1.036–7.815, *p* = 0.042). ACEI/ARB/ARNI therapy also demonstrated a significant protective effect in reducing 3-year mortality (HR: 0.162, 95% CI: 0.036–0.716, *p* = 0.016).

### 3.6. Binary Logistic Regression Analysis for Predictors of Sarcopenia

Table 4 presents the binary logistic regression analysis identifying factors associated with sarcopenia in the study cohort.

In the univariate analysis, female sex was a strong predictor of sarcopenia, with an odds ratio (OR) of 35.000 (95% CI: 7.731–158.456, *p* < 0.001). Age was also significantly associated with sarcopenia (OR: 2.677, 95% CI: 1.116–6.420, *p* = 0.027), while lower weight (OR: 0.376, 95% CI: 0.179–0.788, *p* = 0.010) and height (OR: 0.212, 95% CI: 0.093–0.480, *p* < 0.001) were inversely related to the presence of sarcopenia. CVD was also significantly associated with sarcopenia (OR: 5.359, 95% CI: 1.651–17.393, *p* = 0.005).

In the multivariable analysis, female sex remained a highly significant independent predictor of sarcopenia (OR: 275.748, 95% CI: 17.491–4347.304, *p* < 0.001). Age also retained its strong association (OR: 9.995, 95% CI: 2.150–46.465, *p* = 0.003). However, weight, height, and CVD lost their significance in the multivariable model, indicating that sex and age were the most dominant predictors of sarcopenia in this cohort. Although tricuspid regurgitation (TR III°) showed a trend toward association with sarcopenia in the univariate analysis (*p* = 0.085), this was not statistically significant in the multivariable model.

Overall, the logistic regression analysis highlights that being female and older age are the primary factors independently associated with the presence of sarcopenia in patients undergoing valve surgery for endocarditis.

## 4. Discussion

### 4.1. Sarcopenia as a Relevant Comorbidity in Endocarditis Patients and the Role of Radiology in Its Identification

Sarcopenia, defined by the loss of skeletal muscle mass and strength, has emerged as a significant comorbidity in various medical and surgical conditions, including cardiovascular diseases [10,20], and is particularly impactful in patients undergoing valve surgery for IE [11].

This study highlights sarcopenia as a strong predictor of poor outcomes, with patients in the lowest quartile of PMAi showing significantly higher mortality rates at mid-term follow-up post-surgery. The underlying mechanisms linking sarcopenia to higher mortality are multifactorial, involving frailty, reduced functional capacity, increased postoperative complications, and impaired recovery [21]. Chronic infection, malnutrition, and a catabolic state in IE patients may exacerbate muscle wasting, further weakening these individuals and contributing to their vulnerability [22]. Given the high mortality rates that were observed, it is clear that sarcopenia must be recognized and managed as a significant risk factor in clinical practice.

One of the key strengths of this study is the use of imaging-based assessment of sarcopenia through PMA and its indexation to body surface area (PMAi). Radiology offers a non-invasive method for evaluating sarcopenia, particularly through the use of CT scans, which are often performed to assess embolic events or complications in IE patients. By measuring PMA at the level of the third lumbar vertebra (L3), radiologists can calculate the PMAi, which serves as a reliable indicator of sarcopenia and a robust predictor of mortality with regard to various diseases [23,24,25]. The integration of muscle mass evaluation into routine radiological assessments could prompt earlier interventions, such as nutritional support, prehabilitation, or postoperative rehabilitation, potentially improving outcomes for sarcopenic patients. Thus, radiologists play a crucial role in preoperative risk stratification, helping to identify high-risk patients who may benefit from targeted interventions.

### 4.2. Sarcopenia and Sex: A “Female Problem“?

One typical finding of our study is the strong association between sarcopenia and female sex. In the univariate analysis, women were far more likely to be sarcopenic, with an odds ratio of 35.000. This finding is consistent with the existing literature, for example, in Buckinx et al. [26], where women, especially postmenopausal, are more prone to muscle mass loss due to hormonal changes and other biological factors. Age also played a significant role, with older patients more likely to present with sarcopenia [27]. The combination of older age and female sex appeared to create a particularly vulnerable population [28], underscoring the need for the careful assessment and management of sarcopenia in older women undergoing surgery for IE.

However, our results revealed that while sex was strongly associated with sarcopenia, it did not independently predict mortality. This suggests that while women may be more likely to develop sarcopenia, once sarcopenia is present, sex itself does not significantly influence survival outcomes. Instead, it is the presence of sarcopenia—and its associated factors such as reduced functional capacity and increased frailty—that drives the higher mortality risk. This finding calls into question the common perception of sarcopenia as primarily a “female problem” and suggests that both men and women with sarcopenia require equal attention in clinical settings.

It is important to note that no sex-specific cut-off values for PMAi were deliberately used in this study. This decision was made to avoid over-complicating the analysis and to focus on the general association between sarcopenia and outcomes, irrespective of sex. While sex-specific cut-offs might better reflect physiological differences between men and women, the use of a unified threshold allows for a more straightforward comparison between groups and highlights the overarching impact of sarcopenia on both sexes.

### 4.3. CKI and Mortality

CKI emerged as another critical factor influencing mortality in our cohort regarding the results of Cox hazard regression. Patients with CKI had a significantly higher risk of both 1-year and 3-year mortality, independent of sarcopenia. This is not surprising, given that CKI is well known to increase the risk of adverse outcomes in cardiac surgery due to its association with chronic inflammation, metabolic imbalances, and increased susceptibility to infection [29]. In the context of IE, CKI exacerbates an already complex clinical picture, making surgical intervention riskier and postoperative recovery more challenging.

The interaction between sarcopenia and CKI is worth considering [30]. Both conditions are catabolic states that can compound each other’s effects, leading to further muscle wasting and overall deterioration of health [31,32]. The management of IE in patients with both sarcopenia and CKI presents a unique challenge, requiring a multidisciplinary approach that addresses not only the infection and cardiac complications but also the underlying metabolic and nutritional issues contributing to muscle loss. Early identification and management of these conditions could potentially improve survival outcomes, though further research is needed to explore effective interventions in this high-risk group.

### 4.4. Protective Effect of ACEI/ARB/ARNI Therapy

Our study also identified the use of ACEI, ARB, or ARNI as a protective factor against mortality. Patients on ACEI/ARB/ARNI therapy had significantly lower 1-year and 3-year mortality rates, independent of sarcopenia and other clinical factors. This is consistent with the well-established benefits of these medications in improving cardiac function, reducing afterload, and preventing remodeling in patients with heart failure [33], which is often present in those with IE.

The protective effect of ACEI/ARB/ARNI therapy in our cohort suggests that these medications could be considered as part of the standard management of patients undergoing surgery for IE, particularly in those with heart failure or other cardiovascular comorbidities. Their role in mitigating the adverse effects of sarcopenia, particularly through mechanisms that preserve muscle mass and reduce systemic inflammation, warrants further exploration. So far, only one animal study by Cao et al. [34] can be referred to, which was able to demonstrate reduced bacterial activity in human neutrophil granulocytes under the influence of ACEI. While our study provides compelling evidence for their benefit, prospective trials are needed to confirm these findings and establish more definitive guidelines for the use of ACEI/ARB/ARNI in this patient population.

### 4.5. Clinical Implications and Future Directions

The findings of this study have important clinical implications. Sarcopenia measured by PMAi should be recognized as a major risk factor in patients undergoing valve surgery for infective endocarditis, with the potential to influence survival outcomes significantly. Radiology, specifically through the use of PMAi, offers a practical and reliable method for identifying sarcopenic patients preoperatively. This could help guide clinical decision making, allowing for earlier interventions such as nutritional support or prehabilitation programs aimed at improving muscle mass and strength before surgery.

Given the complex interplay between sarcopenia, CKI, and cardiovascular medications like ACEI/ARB/ARNI, a multidisciplinary approach to managing these patients is crucial. Early identification and aggressive management of sarcopenia, along with optimization of comorbid conditions such as CKI, may improve both short- and long-term survival in this high-risk group. Future studies should focus on exploring the effectiveness of preoperative and postoperative interventions aimed at reversing sarcopenia, as well as investigating the potential synergistic effects of ACEI/ARB/ARNI therapy in these patients.

## 5. Limitations

This study has several limitations that should be acknowledged.

First, the retrospective design may introduce selection bias and limit the ability to establish causal relationships.

Additionally, the sample size, particularly the subgroup of patients with sarcopenia, was relatively small, potentially reducing the statistical power of the analyses.

Another limitation is the use of PMAi as the sole marker of sarcopenia. While PMAi has been validated as a reliable measure of muscle mass, it does not fully capture other aspects of sarcopenia, such as muscle strength and function. The study also lacks detailed nutritional, functional, and lifestyle status assessments [14,16], which could have provided a more comprehensive understanding of sarcopenia’s role and could induce a possible selection bias.

Moreover, the study did not account for the characteristics of vegetations, particularly their embolic potential, which may be a significant prognostic factor in IE. Specifically, the mass motility of vegetations, which can be easily measured by pulsed wave tissue Doppler imaging (PW-TDI), was not considered. Evidence suggests that higher mass peak antegrade velocity is associated with greater embolic risk and, consequently, increased mortality risk in IE patients [35]. Therefore, the absence of this variable may have resulted in an incomplete evaluation of factors influencing mortality in the study population.

Furthermore, the generalizability of the findings may be limited to similar populations and healthcare settings, as the study was conducted at a single tertiary care center.

Finally, while the follow-up period extended to three years, it may still not fully capture the long-term effects of sarcopenia on survival in this patient population.

## 6. Conclusions

This study highlights the significant role of sarcopenia, as measured by PMAi, in predicting short- and mid-term mortality in patients undergoing valve surgery for infective endocarditis. Sarcopenia was associated with increased mortality risk, independent of other clinical factors. Female gender and older age emerged as strong independent predictors of sarcopenia, underscoring the importance of considering these variables when evaluating surgical risk. Given the high mortality rates that were observed in sarcopenic patients, future studies should focus on interventions aimed at improving muscle mass and functional capacity before and after surgery, potentially enhancing outcomes for this high-risk group. These findings support the integration of sarcopenia assessment into preoperative evaluations and clinical decision making to better stratify risk and optimize patient care in endocarditis surgery.

## Figures and Tables

**Table 1:** Psoas muscle area index quartiles. Table 1 categorizes patients into four quartiles (Q1 to Q4) based on their PMAi measured in mm^2^/m^2^.

	PMAi (mm^2^/m^2^)
Q1	<620.96
Q2	620.96–789.60
Q3	789.60–940.91
Q4	>940.91

**Table 2:** Baseline of overall study cohort and of patients with and without sarcopenia.

	Total	Sarcopenia+	Sarcopenia−	*p*-Value
No. (%)				
Total	68 (100.0)	17 (25.0)	51 (75.0)	-
Sex				
Female	20 (29.4)	14 (82.4)	6 (11.8)	<0.001
Male	48 (70.6)	3 (17.6)	45 (88.2)	<0.001
Pre-existing conditions				
Arterial hypertension	35 (51.5)	11 (64.7)	24 (47.1)	0.207
Diabetes mellitus	14 (20.6)	3 (17.6)	11 (21.6)	0.729
COPD	6 (8.8)	3 (17.6)	3 (5.9)	0.139
CKI	8 (11.8)	1 (5.9)	7 (13.7)	0.385
Chronic heart failure	12 (17.6)	2 (11.8)	10 (19.6)	0.463
CVD	24 (35.3)	11 (64.7)	13 (25.5)	0.003
AF	20 (29.4)	7 (41.2)	13 (25.5)	0.219
Premedication				
Diuretics	39 (44.1)	9 (52.9)	21 (41.2)	0.398
Beta-blocker	33 (48.5)	11 (64.7)	22 (43.1)	0.123
ACEI/ARB/ARNI	20 (29.4)	5 (29.4)	15 (29.4)	1.000
Echocardiography				
AR III	15 (22.1)	4 (23.5)	11 (21.6)	0.866
MR III	19 (27.9)	4 (23.5)	15 (29.4)	0.640
TR III	5 (7.4)	3 (17.6)	2 (3.9)	0.060
Preoperative conditions				
Elective surgery	2 (2.9)	2 (11.8)	0 (0.0)	0.013
Urgent surgery	53 (77.9)	13 (76.3)	40 (78.4)	0.866
Emergency surgery	13 (19.1)	2 (11.8)	11 (21.6)	0.373
Intraoperative conditions				
Endocarditis of one heart valve	54 (79.4)	14 (82.4)	40 (78.4)	0.729
Endocarditis of two heart valves	14 (20.6)	3 (17.6)	11 (21.6)	0.729
Endocarditis of three heart valves	0 (0.0)	0 (0.0)	0 (0.0)	1.000
Postoperative conditions				
ECMO	4 (5.9)	1 (5.9)	3 (5.9)	1.000
Bleeding/tamponade	7 (10.3)	0 (0.0)	7 (13.7)	0.107
Stroke	3 (4.4)	2 (11.8)	1 (2.0)	0.088
Valvular complications	1 (1.5)	0 (0.0)	1 (2.0)	0.561
Third-degree atrioventricular block	6 (8.8)	2 (11.8)	4 (7.8)	0.622
Sepsis	1 (1.5)	0 (0.0)	1 (2.0)	0.561
In-hospital death	12 (17.6)	5 (29.4)	7 (13.7)	0.142
Mean ± SD				
Age (years)	62.4 ± 11.9	68.1 ± 10.7	60.5 ± 11.7	0.021
Height (cm)	172.9 ± 8.6	164.8 ± 8.5	175.6 ± 6.8	<0.001
Weight (kg)	81.4 ± 16.2	72.1 ± 11.9	84.4 ± 16.3	0.006
BMI (kg/m^2^)	27.2 ± 4.7	26.6 ± 4.5	27.3 ± 4.9	0.599
BSA (m^2^)	1.9 ± 0.2	1.8 ± 0.2	2.0 ± 0.2	<0.001
PMA (mm^2^)	1590.6 ± 543.9	937.1 ± 130.9	1808.4 ± 444.2	<0.001
PMAi (mm^2^/m^2^)	809.8 ± 245.9	526.7 ± 74.3	904.2 ± 207.1	<0.001
Surgery time (min)	284.9 ± 115.7	287.8 ± 95.1	283.9 ± 122.6	0.904
Clamping time (min)	109.3 ± 56.9	110.0 ± 44.7	109.0 ±60.8	0.952
Perfusion time (min)	167.7 ± 92.6	175.1 ± 76.2	165.2 ± 98.0	0.705
Median ± IQR				
LVEF (%)	55.0 ± 4.0	55.0 ± 7.5	55.0 ± 4.0	0.927

COPD: chronic obstructive pulmonary disease; CKI: chronic kidney injury; CVD: coronary vessel disease; AF: atrial fibrillation; ACEI: angiotensin-converting enzyme inhibitor; ARB: angiotensin receptor blocker; ARNI: angiotensin receptor-neprilysin inhibitor; AR: aortic valve regurgitation; MR: mitral valve regurgitation; TR: tricuspid valve regurgitation; ECMO: extracorporeal membrane oxygenation; BMI: body mass index; BSA: body surface area; PMA: psoas muscle area; PMAi: psoas muscle area index; LVEF: left ventricular ejection fraction.

**Table 3:** Univariate and multivariable cox hazard regression analysis detecting mid-term mortality in dependence of different clinical parameters.

Cox Regression Analysis	Univariate		Multivariable	
	Hazard Ratio (95% CI)	*p*-Value	Hazard Ratio (95% CI)	*p*-Value
1-year mortality				
Gender (female)	3.239 (1.173–8.948)	0.023	2.104 (0.469–9.438)	0.331
Age	0.779 (0.424–1.432)	0.421		
BMI	1.256 (0.797–1.978)	0.326		
BSA	0.831 (0.511–1.352)	0.456		
Arterial hypertension	0.584 (0.208–1.641)	0.307		
Diabetes mellitus	0.910 (0.257–3.224)	0.884		
COPD	1.905 (0.430–8.445)	0.396		
CKI	5.503 (1.867–16.222)	0.002	4.762 (1.452–15.621)	0.010
Chronic heart failure	0.662 (0.149–2.934)	0.587		
CVD	0.913 (0.312–2.671)	0.867		
AF	0.859 (0.273–2.697)	0.794		
LVEF	1.236 (0.739–2.068)	0.418		
AR III°	0.515 (0.116–2.282)	0.382		
MR III°	0.879 (0.280–2.760)	0.825		
TR III°	2.151 (0.485–9.550)	0.314		
Diuretics	2.775 (0.948–8.127)	0.063	2.578 (0.831–7.991)	0.101
Beta blocker	1.226 (0.445–3.381)	0.694		
ACEI/ARB/ARNI	0.149 (0.020–1.136)	0.066	0.112 (0.014–0.870)	0.036
PMA	0.529 (0.291–0.961)	0.036	2.081 (0.232–18.670)	0.513
PMAi	0.494 (0.265–0.920)	0.026	0.378 (0.179–0.795)	0.010
3-year mortality				
Gender (female)	2.124 (0.853–5.289)	0.106		
Age	0.783 (0.453–1.351)	0.379		
BMI	1.338 (0.892–2.007)	0.160		
BSA	0.988 (0.644–1.516)	0.956		
Arterial hypertension	0.498 (0.196–1.265)	0.143		
Diabetes mellitus	0.979 (0.325–2.950)	0.970		
COPD	2.409 (0.701–8.277)	0.163		
CKI	4.224 (1.510–11.818)	0.006	3.097 (1.019–9.409)	0.046
Chronic heart failure	0.801 (0.233–2.748)	0.724		
CVD	1.074 (0.423–2.729)	0.881		
AF	1.086 (0.413–2.858)	0.867		
LVEF	1.122 (0.719–1.753)	0.612		
AINS III°	1.026 (0.434–3.349)	0.720		
MINS III°	0.873 (0.314–2.424)	0.794		
TRINS III°	1.693 (0.391–7.338)	0.482		
Diuretics	3.133 (1.189–8.253)	0.021	2.846 (1.036–7.815)	0.042
Beta blocker	1.881 (0.740–4.780)	0.184		
ACEI/ARB/ARNI	0.235 (0.054–1.019)	0.053	0.162 (0.036–0.716)	0.016
PMA	0.608 (0.366–1.009)	0.054	2.523 (0.465–13.676)	0.283
PMAi	0.541 (0.317–0.925)	0.025	0.457 (0.249–0.841)	0.012

BMI: body mass index; BSA: body surface area; COPD: chronic obstructive pulmonary disease; CKI: chronic kidney injury; CVD: coronary vessel disease; AF: atrial fibrillation; LVEF: left ventricular ejection fraction; AR: aortic valve regurgitation; MR: mitral valve regurgitation; TR: tricuspid valve regurgitation; ACEI: angiotensin-converting enzyme inhibitor; ARB: angiotensin receptor blocker; ARNI: angiotensin receptor-neprilysin inhibitor; PMA: psoas muscle area; PMAi: psoas muscle area index.

**Table 4:** Univariate and multivariable binary logistic regression analysis with regard to the presence of sarcopenia and various clinical parameters.

Binary Logistic Regression	Univariate		Multivariable	
	Odds Ratio (95% CI)	*p*-Value	Odds Ratio (95% CI)	*p*-Value
Sarcopenia (lowest quartile of PMAi)				
Gender (female)	35.000 (7.731–158.456)	<0.001	275.748 (17.491–4347.304)	<0.001
Age	2.677 (1.116–6.420)	0.027	9.995 (2.150–46.465)	0.003
Weight	0.376 (0.179–0.788)	0.010	0.663 (0.226–1.947)	0.455
Height	0.212 (0.093–0.480)	<0.001	1.188 (0.262–5.385)	0.823
BMI	0.863 (0.503–1.480)	0.593		
Arterial Hypertension	2.062 (0.662–6.427)	0.212		
Diabetes mellitus	0.779 (0.189–3.205)	0.730		
COPD	3.429 (0.622–18.908)	0.157		
CKI	0.393 (0.045–3.448)	0.399		
Chronic heart Failure	0.547 (0.107–2.788)	0.468		
CVD	5.359 (1.651–17.393)	0.005	2.016 (0.214–19.004)	0.540
AF	2.046 (0.646–6.482)	0.224		
LVEF	1.191 (0.677–2.095)	0.544		
AR III°	1.119 (0.304–4.123)	0.866		
MR III°	0.738 (0.207–2.636)	0.640		
TR III°	5.250 (0.797–34.585)	0.085	15.733 (0.312–792.195)	0.168
Diuretics	1.607 (0.533–4.846)	0.399		
Beta blocker	2.417 (0.774–7.546)	0.129		
ACEI/ARB/ARNI	1.000 (0.300–3.336)	1.000		

BMI: body mass index; COPD: chronic obstructive pulmonary disease; CKI: chronic kidney injury; CVD: coronary vessel disease; AF: atrial fibrillation; LVEF: left ventricular ejection fraction; AR: aortic valve regurgitation; MR: mitral valve regurgitation; TR: tricuspid valve regurgitation; ACEI: angiotensin-converting enzyme inhibitor; ARB: angiotensin receptor blocker; ARNI: angiotensin receptor-neprilysin inhibitor.

## Data Availability

The data underlying this article will be shared on reasonable request to the corresponding author.

## Supplementary Materials

The following supporting information can be downloaded at: https://www.mdpi.com/article/10.3390/diagnostics14202259/s1, Table S1. Baseline Characteristics of Study Cohort regarding Sex COPD: chronic obstructive pulmonary disease; CKI: chronic kidney injury; CVD: coronary vessel disease; AF: atrial fibrillation; ACEI: angiotensin-converting-enzyme inhibitor; ARB: angiotensin receptor blocker; ARNI: angiotensin receptor-neprilysin inhibitor; AR: aortic valve regurgitation; MR: mitral valve regurgitation; TR: tricuspid valve regurgitation; ECMO: extracorporeal membrane oxygenation; BMI: body mass index; BSA: body surface area; PMA: psoas muscle area; PMAi: psoas muscle area index; LVEF: left ventricular ejection fraction.
