# Supplementary material for: Impact of Psoas Muscle Area Index on Short- and Mid-Term Mortality in Patients Undergoing Valve Surgery for Infective Endocarditis: A Retrospective Analysis
Source: Diagnostics (Basel). 2024 Oct 10;14(20):2259. doi: 10.3390/diagnostics14202259 (PMC11506453; doi:10.3390/diagnostics14202259)
Supplement: Supplementary file 1 [file diagnostics-14-02259-s001.zip › diagnostics-3253480-supplementary.pdf]

	Women	Men	P-value
**No. (%)**			
Total	20 (29.4)	48 (70.6)	-
<u>Pre-existing Conditions</u>			
Arterial Hypertension	10 (50.0)	25 (52.1)	0.876
Diabetes mellitus	3 (15.0)	11 (22.9)	0.462
COPD	2 (10.0)	4 (8.3)	0.825
CKI	3 (15.0)	5 (10.4)	0.593
Chronic Heart Failure	3 (15.0)	9 (18.8)	0.712
CVD	10 (50.0)	14 (29.2)	0.101
AF	7 (35.0)	13 (27.1)	0.514
<u>Premedication</u>			
Diuretics	9 (45.0)	21 (43.8)	0.925
Beta-Blocker	10 (50.0)	23 (47.9)	0.876
ACEI/ARB/ARNI	5 (25.0)	15 (31.3)	0.606
<u>Echocardiography</u>			
AR III°	5 (25.0)	10 (20.8)	0.706
MR III°	6 (30.0)	13 (27.1)	0.807
TR III°	4 (20.0)	1 (2.1)	0.010
<u>Preoperative Conditions</u>			
Elective Surgery	2 (10.0)	0 (0.0)	0.026
Urgent Surgery	15 (75.0)	38 (79.2)	0.706
Emergency Surgery	3 (15.0)	10 (20.8)	0.577
<u>Intraoperative Conditions</u>			
Endocarditis of One Heart Valve	17 (85.0)	37 (77.1)	0.462
Endocarditis of Two Heart Valves	3 (15.0)	11 (22.9)	0.462
Endocarditis of Three Heart Valves	0 (0.0)	0 (0.0)	1.000
<u>Postoperative Conditions</u>			
ECMO	1 (5.0)	3 (6.3)	0.842
Bleeding/Tamponade	1 (5.0)	6 (12.5)	0.354
Stroke	1 (5.0)	2 (4.2)	0.879
Valvular Complications	0 (0.0)	1 (2.1)	0.516
Third-Degree Atrioventricular Block	2 (10.0)	4 (8.3)	0.825
Sepsis	0 (0.0)	1 (2.1)	0.516
In-Hospital Death	6 (30.0)	6 (12.5)	0.085
**Mean ± SD**			
Age (years)	60.3 ± 14.6	63.2 ± 10.5	0.419
Height (cm)	163.7 ± 5.8	176.8 ± 6.3	<0.001
Weight (kg)	71.5 ± 12.3	85.5 ± 15.9	0.001
BMI (kg/m^2)	26.7 ± 4.8	27.3 ± 4.8	0.651
BSA (m^2)	1.8 ± 0.1	2.0 ± 0.2	<0.001
PMA (mm^2)	1074.5 ± 311.9	1805.6 ± 471.0	<0.001
PMAi (mm^2/m^2)	602.9 ± 142.4	896.1 ± 228.4	<0.001
Surgery Time (min)	283.6 ± 99.1	285.4 ± 122.9	0.952
Clamping Time (min)	107.1 ± 47.8	110.2 ± 60.7	0.837
Perfusion Time (min)	173.4 ± 73.5	165.3 ± 100.1	0.744

Median ± IQR			
LVEF (%)	55.0 ± 3.8	55.0 ± 4.0	0.908

Supplementary Table S1. Baseline Characteristics of Study Cohort regarding Sex

COPD: chronic obstructive pulmonary disease; CKI: chronic kidney injury; CVD: coronary vessel disease; AF: atrial fibrillation; ACEI: angiotensin-converting-enzyme inhibitor; ARB: angiotensin receptor blocker; ARNI: angiotensin receptor-neprilysin inhibitor; AR: aortic valve regurgitation; MR: mitral valve regurgitation; TR: tricuspid valve regurgitation; ECMO: extracorporeal membrane oxygenation; BMI: body mass index; BSA: body surface area; PMA: psoas muscle area; PMAi: psoas muscle area index; LVEF: left ventricular ejection fraction
